# 

**Published:** 1908-11-21

**Authors:** 


### BOOKS RECEIVED.

Macmillan and Co.

"Hygiene for Nurses." By Isabel McIsaac

Stanley, Paul and Co.

"Priests of Progress." By G. Colmore.

Baillière, Tindall, and Cox.

"Physiological Principles in Treatment." By W. Langdon Brown, M.D.

W. Blackwood and Sons.

"The Bias." By Marguerite Curtis.

Cassell and Co.

"Diseases of the Skin." By Sir Malcolm Morris, K.C., V.O. New and enlarged edition.

J. B. Lippincott Co.

"Clinical Diagnosis." By C. P. Emerson, M.D.

John Murray.

"Intracellular Enzymes." By H. M. Vernon, M.A., M.D.

George Newnes, Ltd.

"Every Mother's Baby Book." By Dr. Andrew Wilson.

The Scientific Press, Ltd.

"Home Nursing of Sick Babies." By T. M. Brewster.

THE
### GRADUATES' MEDICAL SCHOOLS.

DIARY FOR WEEK NOV. 23 to NOV. 27.

**THE POST-GRADUATE COLLEGE, West London Hospital, Hammersmith, W.**

**At 10 a.m.**
**Nov. 23 and 26,** Mr. Etherington Smith, **Demonstration of Surgical Cases.**

**At 12 noon.**
**Nov. 23,** Dr. Low, **Pathological Demonstration.**

**At 12.15 p.m.**
**Nov. 25 and 27,** Dr. Pritchard, **Practical Medicine.**

**At 5 p.m.**
**Nov. 23,** Dr. Saunders, **Clinical, I.**
**Nov. 24,** Dr. Low, **Plague.**
**Nov. 25,** Dr. Beddard, **Medicine, IV.**
**Nov. 26,** Mr. Baldwin, **Surgery, IV.**
**Nov. 27,** Dr. Hood, **Some Clinical Observations on Heart Disease.**

**ST. JOHN'S HOSPITAL FOR DISEASES OF THE SKIN, Leicester Square, W.C.**

**At 6 p.m.**
**Nov. 26,** Chesterfield Lectures, **Acne Vulgaris.**

**MOUNT VERNON HOSPITAL, Fitzroy Square.**

**At 5 p.m.**
**Nov. 26,** Dr. S. W. Price, **Demonstration of Cases of Heart Disease.**

**THE HOSPITAL FOR SICK CHILDREN, Great Ormond Street, W.C.**

**At 4 p.m.**
**Nov. 26,** Dr. Colman, **A Demonstration on Selected Medical Cases.**

**THE WEST-END HOSPITAL FOR NERVOUS DISEASES, Welbeck Street, W.**

**At 5 p.m.**
**Nov. 26,** Mr. Cantlie, **Spinal Cord Surgery.**

**THE THROAT HOSPITAL, Golden Square, W.**

**At 5.30 p.m.**
**Nov. 23,** Mr. Parker, **Treatment of Disease of the Accessory Sinuses of the Nose.**
**Nov. 26,** Mr. Rose, **Rhinitis Sicca, Rhinitis Atrophica.**

**NATIONAL HOSPITAL FOR THE PARALYSED AND EPILEPTIC, Queen Sq., Bloomsbury, W.C.**

**At 3.30 p.m.**
**Nov. 24,** Mr. Sargent, **Surgery of the Nervous System.**
**Nov. 27,** Mr. Leslie Paton, **Optic Neuritis.**

**NORTH-EAST LONDON POST-GRADUATE COLLEGE, Prince of Wales's General Hospital, Tottenham, N.**

**At 4.30 p.m.**
**Nov. 26,** Dr. A. J. Whiting, **The Peroneal Type of Muscular Atrophy.**

**MEDICAL GRADUATES' COLLEGE AND POLYCLINIC, 22 Chenies Street, W.C.**

**At 5.15 p.m.**
**Nov. 23,** Mr. Yearsley, **Stammering.**
**Nov. 24,** Dr. Luff, **The Use of Calcium Salts in various Morbid Conditions.**
**Nov. 25,** Dr. Purves Stewart, **Myopathies.**
**Nov. 26,** Dr. Perkins, **Angina Pectoris: its Diagnosis, Treatment, and Prognosis.**

**LONDON SCHOOL OF CLINICAL MEDICINE, Seamen's Hospital, Greenwich, S.E.**

**At 3.15 p.m.**
**Nov. 23,** Mr. Wm. Turner, **The late Complications of Appendicitis.**

**At 3.30 p.m.**
**Nov. 25,** Mr. Cargill, **Syphilitic Eye Affections.**

### THE MEDICAL INSPECTION OF SCHOOL CHILDREN.

A final course of lectures and demonstrations on the Medical Inspection of School Children will be given by Dr. James Kerr, the Chief Medical Officer of the Education Department of the London County Council, on January 11, 12, 13, and 14, 1909. The lectures will be delivered in the Rooms of the Society of Medical Officers of Health, and the demonstrations will take place in the Special Schcols of the London County Council. The course will also include lectures by Drs. C. J. Thomas and A. H. Hogarth, of the London County Council Education Department; Dr. H. Meredith Richards, Messrs. Macleod Yearsley, C. Edward Wallis, and others. The subjects will comprise General Medical Inspection of School Children, Special Medical Inspection, Eyes, Ears, Teeth, Infectious Diseases, Defective Children, School and Home, Office Routine, and the Preparation of Reports. The course is open to all medical graduates, and those desirous of attending should make early application to Mr. Wm. A. Lawton, at the Offices of the Society of Medical Officers of Health, 1 Upper Montague Street, Russell Square, London, W.C., as accommodation is only available for a limited number.

